# Subcellular structural plasticity caused by the absence of the fast Ca^2+^ buffer calbindin D-28k in recurrent collaterals of cerebellar Purkinje neurons

**DOI:** 10.3389/fncel.2014.00364

**Published:** 2014-11-05

**Authors:** David Orduz, Alain Boom, David Gall, Jean-Pierre Brion, Serge N. Schiffmann, Beat Schwaller

**Affiliations:** ^1^Laboratory of Neurophysiology, UNI, Université Libre de Bruxelles (ULB)Bruxelles, Belgium; ^2^Laboratory of Histology, Neuroanatomy and Neuropathology, UNI, Université Libre de Bruxelles (ULB)Bruxelles, Belgium; ^3^Anatomy, Department of Medicine, University of FribourgFribourg, Switzerland

**Keywords:** calcium buffer, calbindin D-28k, Cerebellum, axonal recurrent collateral, synaptic cleft, synaptic bouton

## Abstract

Purkinje cells (PC) control spike timing of neighboring PC by their recurrent axon collaterals. These synapses underlie fast cerebellar oscillations and are characterized by a strong facilitation within a time window of <20 ms during paired-pulse protocols. PC express high levels of the fast Ca^2+^ buffer protein calbindin D-28k (CB). As expected from the absence of a fast Ca^2+^ buffer, presynaptic action potential-evoked [Ca^2+^]_i_ transients were previously shown to be bigger in PC boutons of young (second postnatal week) CB-/- mice, yet IPSC mean amplitudes remained unaltered in connected CB–/– PC. Since PC spine morphology is altered in adult CB–/– mice (longer necks, larger spine head volume), we summoned that morphological compensation/adaptation mechanisms might also be induced in CB–/– PC axon collaterals including boutons. In these mice, biocytin-filled PC reconstructions revealed that the number of axonal varicosities per PC axon collateral was augmented, mostly confined to the granule cell layer. Additionally, the volume of individual boutons was increased, evidenced from z-stacks of confocal images. EM analysis of PC–PC synapses revealed an enhancement in active zone (AZ) length by approximately 23%, paralleled by a higher number of docked vesicles per AZ in CB–/– boutons. Moreover, synaptic cleft width was larger in CB–/– (23.8 ± 0.43 nm) compared to wild type (21.17 ± 0.39 nm) synapses. We propose that the morphological changes, *i.e.,* the larger bouton volume, the enhanced AZ length and the higher number of docked vesicles, in combination with the increase in synaptic cleft width likely modifies the GABA release properties at this synapse in CB–/– mice. We view these changes as adaptation/homeostatic mechanisms to likely maintain characteristics of synaptic transmission in the absence of the fast Ca^2+^ buffer CB. Our study provides further evidence on the functioning of the Ca^2+^ homeostasome.

## INTRODUCTION

Activity-dependent synaptic plasticity, *i.e.,* modulation of the synaptic strength of two neurons coupled by chemical synapses is the hallmark of most synapses (Kim and Linden, 2007). Induction of plasticity occurs at highly variable time scales. Such changes may be (*i*) very brief and restricted to the time period from the start to the end of trains of APs and are named STP, (*ii*) longer lasting as in the case of Hebbian-type plasticity including LTP or depression (LTP and LTD, respectively), or (*iii*) operating at time scales of hours to days and the process is referred to as HSP (Turrigiano, 2012). The latter is thought to maintain network stability by fine-tuning global synaptic strength under conditions when its activity diverges from tolerant (stable) physiological levels, *e.g.,* as the consequence of an insult, chronic suppression of activity or mutations in genes implicated in synaptic transmission. Many different processes occurring in the pre- and/or postsynaptic compartment have been described for the various types of plasticity (Zucker and Regehr, 2002; Blitz et al., 2004; Vitureira and Goda, 2013). Since transmitter release at presynaptic terminals is a Ca^2+^-dependent process, the precise shape of Ca^2+^ signals within presynaptic terminals is a critical determinant (Cavazzini et al., 2005). Among other components linked to Ca^2+^ entry and extrusion, Ca^2+^ buffers are considered as relevant modulators of these presynaptic Ca^2+^ signals. Examples of such buffers characterized by either slow or fast Ca^2+^-binding kinetics include parvalbumin (PV) and CB, respectively (Schwaller, 2010). Both of these proteins were previously shown to modulate STP (Blatow et al., 2003; Collin et al., 2005; Orduz et al., 2013). The quantitative aspects of a presynaptic Ca^2+^ signal then determine the time course and amount of neurotransmitter released into the synaptic cleft. This, in turn, leads to an appropriate response in the postsynaptic neuron, in the form of an inhibitory or excitatory postsynaptic response, depending on the type of neurotransmitter and on the type(s) of receptors. Also at the postsynaptic side, several mechanisms leading to modulation of synaptic transmission have been described and include receptor saturation, receptor desensitization, receptor distribution/clustering and/or phosphorylation (von Gersdorff and Borst, 2002), but also morphological changes (*e.g.,* spine shape; Alvarez and Sabatini, 2007). Thus, while pre- and postsynaptic compartments show a large degree of plasticity, the architecture of the synaptic cleft, most notably cleft width is considered as relatively resistant to plasticity. The cleft width is assumed to be essentially determined by interactions of proteins anchored in the pre- and postsynaptic membrane including neurexin family members and neuroligins, respectively. Consequently, the cleft width for a particular synapse shows extremely little variation, most often less than 5%, *i.e.,* 0.4 nm variation for a “typical” synapse cleft width of approximately 20 nm, as modeled based on Monte Carlo simulations (Savtchenko and Rusakov, 2007) or measured from EM images. Structural changes with respect to synaptic cleft width, dimension of the AZ/PSD, curvature and presynaptic bouton volume have been reported. These changes are often the result of short- or long-term pathological insults such as oxygen/glucose deprivation (OGD; Lushnikova et al., 2011), long-term exposure to lead (Han et al., 2014), aluminum (Jing et al., 2004) or biphenyl-A, a presumed endocrine disruptor (Xu et al., 2013a,b). But also exposure of ovarectomized (OVX) rats to estradiol benzoate increases the synaptic cleft width of CA1 hippocampal synapses in the pyramidal cell layer (Xu and Zhang, 2006). Most recently, a change in the synaptic cleft architecture was reported in mice deficient for the neurexin family member contactin associated protein-like 4 (CNTNAP4), a protein implicated in autism spectrum disorders (ASD; Karayannis et al., 2014). Based on the observation that the absence of the fast Ca^2+^ buffer CB in presynaptic boutons of PC recurrent axon collaterals of PN7–PN12 mice had no effect on either basic synaptic transmission (mean IPSC amplitude) or on paired-pulse facilitation (PPF; Bornschein et al., 2013), we summoned adaptive/homeostatic mechanisms to be present at this synapse and focused on changes in the synapse morphology. We observed an increase in presynaptic bouton volume, paralleled by an increase in the AZ/PSD length, in the number of docked vesicles and moreover, an increase in synaptic cleft width. This is in line with the proposition of Loebel et al. (2013) that “ongoing synaptic plasticity results in matched presynaptic and postsynaptic modifications, in which elementary modules that span the synaptic cleft are added or removed as a function of experience.”

## MATERIALS AND METHODS

### ANIMALS

The experiments and procedures conformed to the regulations of the Institutional Ethical Committee of the School of Medicine of the Université Libre de Bruxelles, Belgium and the University of Fribourg, Switzerland. In this study we used C57Bl/6J mice as control animals and null-mutant mice for (CB–/– mice; Airaksinen et al., 1997).

### CB IMMUNOHISTOCHEMISTRY AND WESTERN BLOT ANALYSES

To assess developmental CB expression in the cerebellum, immunohistochemistry with CB antibodies was performed with sections from mice of the same litters (PN5–PN25). Animals were perfused intracardially with phosphate-buffered saline (PBS), followed by paraformaldehyde (4%) and rinsed with PBS (0.1 M). Cerebella were removed and placed overnight in a 4% PFA solution. Sagittal slices of the cerebellar vermis (80 μm) were prepared in ice-cold PBS solution (4°C). Slices from animals belonging to the same litter were processed simultaneously. Primary antibody incubations were performed at 4°C for 24 h, using rabbit anti-CB 38a antiserum (1:500, Swant, Marly, Switzerland) and secondary antibody incubations for 1 h at room temperature with Alexa Fluor-labeled donkey anti-rabbit 488 (1:500, Invitrogen).

For Western blot analyses, mice from similar age groups (PN6–PN25; *n* = 3 animals for each age group/series, 2 series for all but one for the PN25 group) were deeply anesthetized by CO_2_ inhalation and perfused transcardially with ice-cold PBS solution (4°C). Dissected cerebellum were homogenized in 10 mM Tris-HCl/1 mM EDTA, pH 7.4. Soluble protein fractions (supernatant) were obtained by centrifugation of homogenates at 15,000 × g for 30 min. Proteins (1 μg) were separated by SDS-PAGE (12%) and transferred on nitrocellulose membranes. Membranes were incubated with a blocking solution (LI-COR Biosciences GmbH, Bad Homburg, Germany) for 1 h. Next, they were incubated overnight with primary antibodies against CB (CB 38a; dilution, 1:1000; Swant) or against α-actin (mouse monoclonal, dilution 1:1000; Sigma). As secondary antibodies we used either anti-rabbit labeled with IRDye 800CW or anti-mouse labeled with IRDye 680RD (dilution 1:10,000, incubation for 1 h). The bands corresponding to CB and α-actin were visualized and quantified by the Odyssey®; Infrared Imaging System (LI-COR) and the corresponding software, respectively.

### BIOCYTIN-FILLING OF PC AND SYNAPTOPHYSIN LABELING

Sagittal cerebellar slices (180 μm) were prepared from WT and CB–/– mice (PN18–PN25). Animals were anesthetized with halothane before decapitation. After rapid removal of the cerebellum, slices were cut with a Leica VT1000S vibratome (Leica Microsystems), in ice-cold bicarbonate-buffered saline (BBS) at 4°C, containing (in mM): 125 NaCl, 2.5 KCl, 1.25 NaH_2_PO_4_, 26 NaHCO_3_, 2 CaCl_2_, 1 MgCl_2_, 10 glucose and equilibrated with a 95%O_2_–5%CO_2_ mixture (pH 7.3). Before experiments, slices were incubated at 34°C for 45 min in the same saline. After this period, a single slice was transferred to a recording chamber and submerged in continuously flowing BBS at 22–24°C with a flow rate of 1.5 ml/min.

To increase the probability of finding PC with intact axons and recurrent collateral arbors, we targeted PC located (*i*) in the second/third somata below the surface of the slice and (*ii*) in flat regions between the apex and base of a lobule. These regions were clearly less damaged during the slicing procedure. PC were visualized with a 63x water immersion objective placed in a Zeiss upright microscope (Axioskop 2FS Plus, Zeiss) and recorded using the whole-cell configuration of the patch-clamp technique (WCR) with a double EPC-10 operational amplifier (Heka Elektronik). Patch pipettes were made from borosilicate glass capillaries (Hilgenberg GmbH) with a two-stage vertical puller (PIP 5, Heka Elektronik) with resistances between 3.5 and 5 MΩ. The intracellular solution contained the following (in mM): 150 K-gluconate, 4.6 MgCl_2_, 10 K-Hepes, 0.1 K-EGTA, 0.4 Na-GTP, 4 Na-ATP, and 0.1 CaCl_2_ (pH 7.2) and 0.4% biocytin.

WCR was maintained for at least 20 min after break-in to improve labeling up to distal regions of recurrent collaterals. Once this time period had elapsed, high-resistance outside-out patches were obtained during pipette withdrawal. An extra time period of 15 min was allotted for biocytin diffusion, then slices were fixed overnight in 4% PFA (4°C) and rinsed with PBS (0.1 M). For double immunostainings we first incubated slices with a primary rabbit anti-synaptophysin 1 antibody (1:200, Synaptic systems) for 24 h, followed by secondary anti-rabbit 633 antibodies (1:500, Invitrogen) and streptavidin-conjugated NL557 (1:5000, R&D systems) at room temperature during 1 h.

### CONFOCAL MICROSCOPY AND IMAGE ANALYSIS

Confocal acquisitions were obtained by using an Axiovert 200M-LSM 510 META microscope (Zeiss) equipped with a Plan-Neofluar 10x/0.3 W or a C-Apochromat 40x/1.2 W objective. We used three laser beams (a 488 nm argon and 543 and 633 nm helium–neon laser lines) with filters to selectively detect emitted fluorescence from CB-positive regions (BP 500–550 nm), biocytin-filled cells (BP 565–615 nm) and synaptophysin-positive zones (BP 650–710).

For the CB quantification during PN development by immunofluorescence acquisition, parameters were optimized for non-saturated CB signals at PN20 on 60 μm-thick z-stacks composed of 115 × 115 μm images with a z-step of 3 μm. Subsequently similar stacks for sections from younger mice from the same litter were acquired using the identical parameters. Because fluorescence can be artificially reduced on optical slices as the result of incomplete antibody penetration, we used ImageJ software (http://imagej.nih.gov/ij/) to identify the five images within each stack with the strongest fluorescence intensities. A maximal intensity z-projection was made with these five selected optical slices and we calculated the mean fluorescence values of the PC layer as percentage of fluorescence normalized to PN20. For colocalization studies, the fluorescence intensity profiles were obtained from single optical sections of a PC after biocytin-filling (green) and synaptophysin labeling (red).

For the localization of PC boutons in the different PC layers, biocytin-loaded PC were reconstructed from an area of 350 × 350 μm and consisting of 80 z-sections (0.8 μm each). To visualize recurrent collateral boutons on PC somata, z-stacks containing 30 images (thickness of 0.4 μm per section) and covering a region of 14 × 14 μm were selected. We applied a median filter to each z-projection from the stacks to reduce noise and to perform morphology measurements (*e.g.,* bouton volume) with ImageJ tools. To assess the distribution pattern of boutons from several PC, all PC somata were superimposed and axonal arbors were oriented in such a way as to respect their position within cerebellar layers and to position the main axon path toward the white matter. We then determined x and y positions for each bouton. These coordinates were used to build a 2D-matrix in MatLab environment permitting to quantify the number of boutons within 20-μm side length squares. This size was chosen, since it corresponds approximately to the diameter of a PC soma. We then calculated the bouton density as the number of boutons per μm^2^ and used a color gradient scale (heat map) to visualize regions with high and low bouton densities. For bouton volume measurements, we first defined the “boundaries of a bouton” along the axon recurrent collateral as the points at which collaterals dilate to twice its axonal diameter. Next we counted the number of fluorescent voxels between these points (or in the case of terminal boutons only from the starting point). Because a single voxel corresponded to a cube of 0.00004 μm^3^ (0.01 × 0.01 × 0.4 μm for x, y, and z dimensions), we were able to calculate the approximated value of bouton volumes. Changes in volume were more evident when we performed 3D-surface reconstructions of each bouton with Osirix software, as it is shown in **Figure 3D**.

### ELECTRON MICROSCOPY AND ULTRASTRUCTURAL MEASUREMENTS

Mice (PN20) were anaesthetized and were perfused transcardially with a heparinized saline solution followed by fixative containing 4% PFA and glutaraldehyde (0.25%) in 0.1 M phosphate buffer (pH 7.3). Brains were removed, cerebella were dissected and further fixed for 4 h in the same fixative. For pre-embedding immunolabeling, cerebella were sagittally sectioned on a vibrating blade microtome (Ted Pella, Inc., USA) at a thickness of 35–40 μm. The sections were processed for immunolabeling with the streptavidin–biotin-peroxidase complex (ABC, Vector, UK). In brief, the tissue sections were pre-incubated for 1 h in 5% normal goat serum with 0.5% Triton X-100 and then incubated for 24 h at 4°C with TBS containing anti-L7 polyclonal rabbit antibody (1:500, Santa Cruz). After washing in TBS, sections were incubated for 4 h with the goat anti-rabbit antibody conjugated to biotin (1:100, Vector, UK) followed by the streptavidin–biotin-peroxidase complex (Vector). The peroxidase activity was revealed using diaminobenzidine as chromogen (DAB; Dako, Belgium). 35–40 μm-thick cerebellar slices were then observed with transmitted light on an inverted microscope (Zeiss, Axiovert 200 M), which corroborated L7-labeling on PC somata and PC collateral boutons from both WT and CB–/– mice. After washing in Millonig’s buffer with 0.5% (w/v) sucrose for 24 h, sections were post-fixed in 2% (w/v) OsO_4_ for 30 min, dehydrated in a graded series of ethanol and embedded in Epon-resin LX112 (Ladd Research Industries, Inc., USA). Semi-thin sections were stained with toluidine blue. Ultrathin sections (30 nm) collected on nickel grids were stained in 2% aqueous uranyl acetate and lead citrate and observed with a Zeiss EM 809 microscope at 80 kV coupled to a digital camera (Jenoptik, Prog Res C14, Germany).

PC – PC contacts were found by careful inspection of PC somata. PC slightly labeled by L7 antibodies presented a better ultrastructure, which facilitated the extraction of morphological parameters. In contrast, strong labeling hid the most important details by obscuring the synapses. Thus, we focused our analyses on morphologically well preserved, lightly stained PC *e.g.,* shown in **Figure 4B**.

Morphological measurements were made with ImageJ tools on EM images at x 30,000 magnifications. To analyze PC–PC synapses, we first identified PC soma based on their characteristic soma shape and size. The PC soma was surveyed under high magnification and PC–PC synapses were recognized by the presence of (*i*) L7-labeling, (*ii*) symmetrical membrane appositions, (*iii*) pleomorphic synaptic vesicles, and (*iv*) eventually its myelinated axon, when the slicing procedure preserved it. We counted the number of AZ per bouton and their individual lengths. Only synapses that had a clear synaptic cleft separating the pre- and postsynaptic elements were taken for width measurements. These measurements required a more standardized method, because the synaptic cleft boundaries are not really well defined when measuring it on EM images. A true consensus is missing on how to reduce the observer’s bias in measurements of “the real cleft width.” It is often defined as the brightest region between pre and postsynaptic membranes and usually measured as the distance between pre- and postsynaptic electro-dense (black) peaks. However, this procedure can lead to mistakes, because those peaks can vary significantly from one synapse to the other.

To solve this problem we adapted the following method consisting of three steps: (1) on digital-zoomed images, we traced five 100 nm-length straight lines from the pre- to the postsynaptic side, perpendicular to the membranes. These lines were separated from each other by 10-nm intervals; (2) we extracted the intensity profiles for each line and averaged them. This reduced the noise to better resolve the valley between peaks that is considered to be the real synaptic width; and (3) we identified the point at which the intensity of the signal decreased by 10% from the presynaptic peak to the minimal signal value into the valley (as it is shown by the gray dotted line in **Figure 4C**) and similarly for the postsynaptic peak with respect to the minimal value into the valley. The distance between both were assumed to represent the real synaptic width. We found that our method is more robust and less biased compared to measurements by experimenter’s eye-inspection as demonstrated by manual-blind measurements *vs.* the standardized method (see Figure S1).

### DATA ANALYSIS AND STATISTICS

Data analyses were performed using Neuromatic software package and custom routines within the IgorPro (Wavemetrics, Lake Oswego, OR, USA) or Matlab environment. All values are expressed as mean ± SEM. Student’s *t*-test were performed within the Excel software package (Microsoft). The significance level was established at *P* < 0.05 (*). Cumulative distributions were compared using the Kolmogorov–Smirnov test. A homoscedasticity test was used to determine whether measurements of synaptic cleft width within a group have equal variances or not (separately for either WT or CB–/–). This was important to justify that the homeostatic rearrangement in synaptic cleft values are indeed genotypically stable changes (low variability for each genotype, independent of the number of samples). For this the test initially consists of calculating the ratio of the largest to the smallest of several sample variances for a genotype (F_max_). If this ratio is equal to unit, then the null hypothesis is accepted (variances are not different), but this was not the case for both genotypes. In that case, one needs to know how high F_max_ may be for each genotype before to accept the null hypothesis. This is given by a statistical value called critical-F_max_ = F_max_ α[k, n-1], where α = 0.05; *k* = number of mice; *n* = number of observations per mouse. The critical-F_max_ for both genotypes, obtained from statistic tables (David, 1952), presented higher values compared to F_max_ for each genotype, demonstrating that variances are equal within each genotype group. Coefficients of variation were calculated as the square root of the ratio of SD to mean. The box-and-whiskers graphs were performed using Prism 4.0 (GraphPad software) and show the median (the line in the middle) and the 25th – 75th percentile (the box extension).

## RESULTS

### DEVELOPMENTAL INCREASE IN CB EXPRESSION IN MOUSE CEREBELLAR PC REACHING A PLATEAU AT PC MATURITY

**FIGURE 1:**
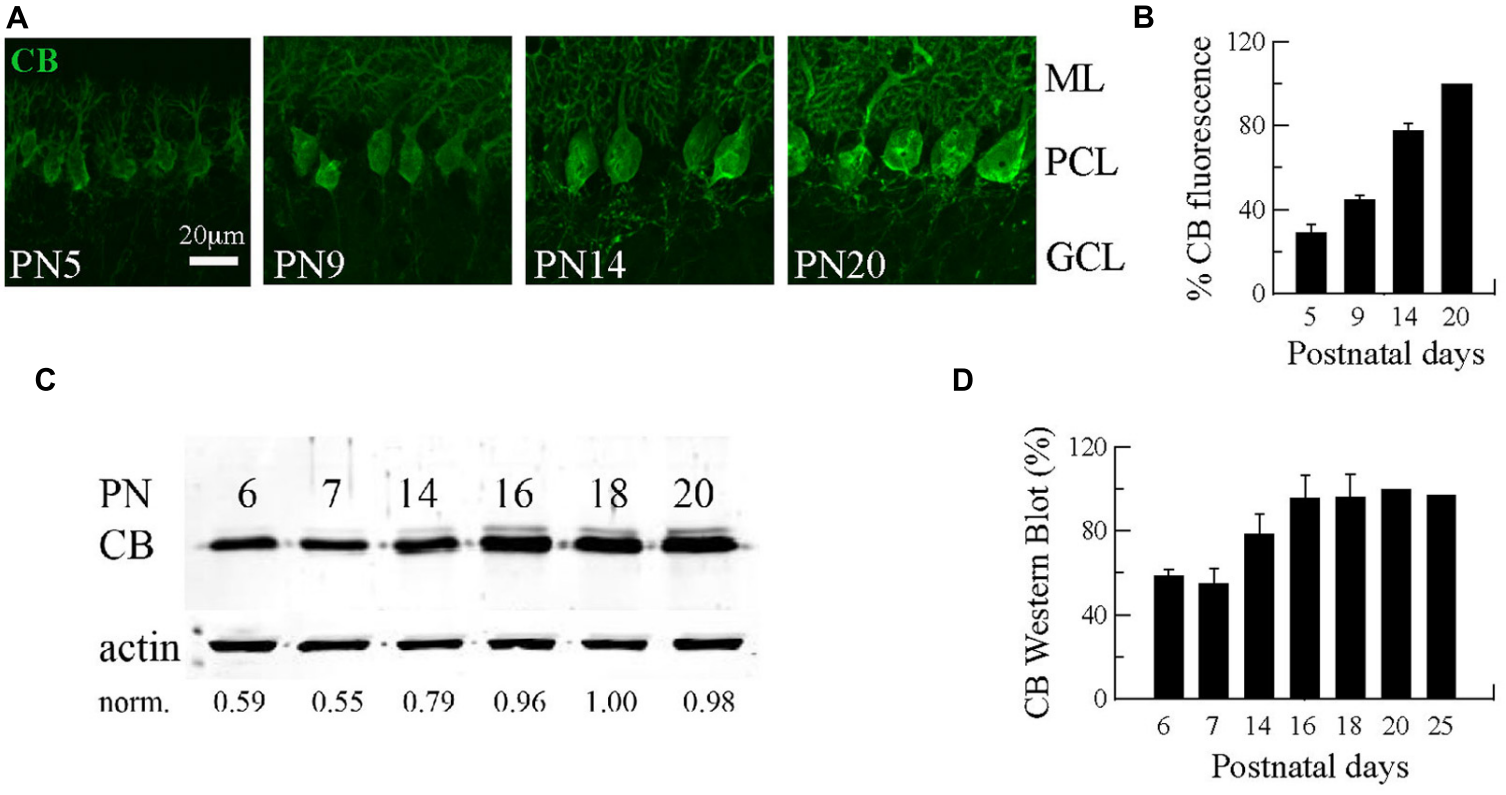
**Developmental regulation of calbindin-D28k (CB) expression in mouse cerebellar PC. (A)** Confocal images of the cerebellar cortex at different ages from PN5–PN20 on sagittal slices treated with anti-CB antibodies. The expression of CB is detected early during the first PN week in dendrites, somata and axons of PC. The intensity of IHC signals increases progressively until PN20. **(B)** Mean CB fluorescence values of the PC layer at different ages (*n* = 3 mice per age). Data are normalized to PN20. **(C)** Age-dependent increase in the total CB protein expression levels from PN6–PN20 determined by quantitative Western blots (average from 2 independent experiments, *n* = 3 mice per time point). The actin signal was used for normalization (norm.). In each of the 2 experiments, the normalized signal at PN20 was set as 1.00. **(D)** Pooled data for Western-blots of cerebella from PN6–PN25 (*n* = 3 mice per age) reached a plateau in CB expression at the end of the third week (ML, molecular layer; PCL, Purkinje cell layer, GCL, granule cell layer).

The first three postnatal (PN) weeks are critical for the establishment of a mature cerebellar circuit in rodents (van Welie et al., 2011). Only at the end of the second PN week PC axon collateral arbors stabilize their length, spatial distribution and number of axonal boutons, a process probably achieved by cerebellar myelination that limits the expansion of axon collaterals and conserves final and stable synapses (Gianola et al., 2003). CB has been commonly used as “the prototypical PC marker,” but little attention has been paid to the temporal aspect of CB expression during this critical period of cerebellar development. With this aim, we quantified CB expression levels in the cerebellum of WT mice by immunofluorescence and Western blot analyses from PN5 to PN20 (**Figure 1**). Cerebellar slices at different ages were processed simultaneously and values for the two types of measurements were normalized to PN20 levels. Slices obtained from younger animals (PN5–PN14) were imaged with the same optical settings (see Materials and Methods). CB immunofluorescence was detected at the first PN week (PN5) and the staining intensity increased progressively until PN20 (**Figure 1A**). Semi-quantification of CB expression measured by the intensity of fluorescence in the PC layer indicated a developmental upregulation of CB (**Figures 1A,B**). As a second method to quantify the developmental increase in CB expression levels, we performed Western blot analyses with two additional time points that confirmed the increase in CB expression during that period; a plateau was reached during the third PN week (**Figures 1C,D**). The combined results from immunofluorescence and Western blot analyses indicated an approximately twofold increase in CB expression levels taking place between the end of the first and the end of the third PN week. This strongly indicates that morphological stabilization of PC axon collaterals and CB-regulated intracellular Ca^2+^-signaling processes in PC recurrent collaterals, as well as in the soma and dendrites are likely to be temporally coordinated. We then focused our further investigation on mice from PN18–PN25, time points when CB expression levels had attained steady-state levels, thus all further experiments were carried out with animals of this age range.

### TERMINAL BOUTONS OF PC COLLATERALS ON NEIGHBOR PC ARE SYNAPSES AND CO-LOCALIZE WITH CB

**FIGURE 2:**
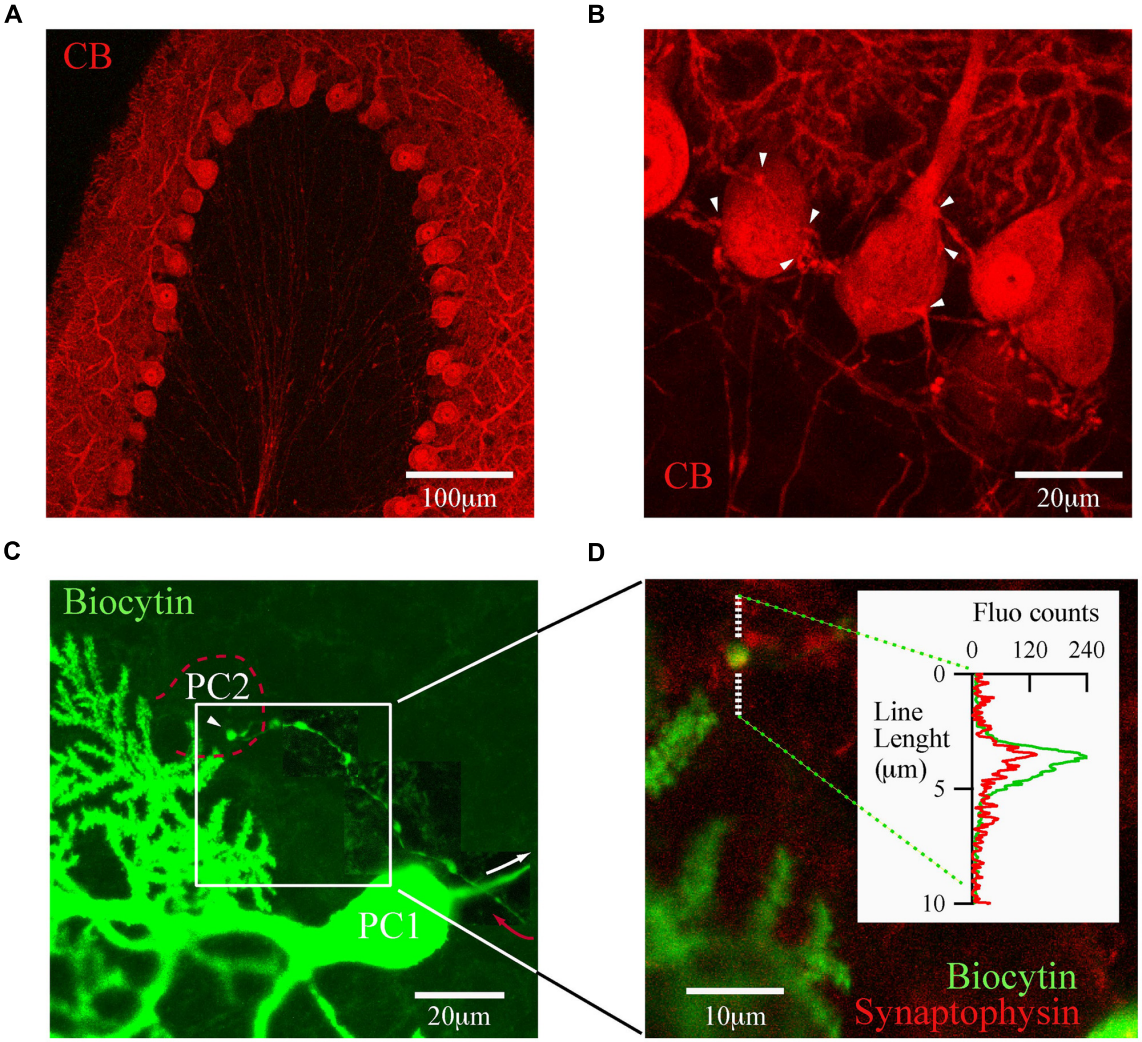
**calbindin D-28k labels presynaptic PC collateral boutons onto neighbor PC. (A)** Confocal image of a cerebellar lobule showing that in the cerebellar cortex, CB expression is restricted to PC (PN18, sagittal slice). **(B)** At high resolution, CB labels the recurrent axon collateral plexus and their boutons on PC somata (white arrowheads). **(C)** Confocal image of one biocytin-filled PC (PC1; green)) in which the main axon (white arrow) gives rise to a recurrent collateral that returns to the PC layer (red arrow) and terminates on a neighbor PC soma (PC2, dotted red line). To reveal the axon collateral, the contrast was adapted for three adjacent areas of the final stack. **(D)** Zoom of the white square in **(C)** shows a biocytin-loaded bouton (green) on the PC layer (white square) co-localizing with synaptophysin (red). Line analysis (inset) confirmed that boutons on PC layer are *bona fide* PC–PC synapses.

The trilaminar architecture of the cerebellar cortex is easily distinguished by CB immunolabeling of a cerebellar lobule (**Figure 2A**). PC dendrites, somata, and axons determine the boundaries of the molecular, PC and granule cell layers, respectively. This labeling also distinguishes recurrent collateral arbors and their terminal boutons on neighbor PC (**Figure 2B**). The strong CB immunofluorescence of PC somata makes it difficult to resolve presynaptic boutons, a requirement for detailed morphological analyses of PC–PC connections. To surmount this difficulty we loaded PC with biocytin via the patch pipette during whole cell recordings in PN18–25 mice. This approach permitted us to identify from a single PC the following structures: the main axon, recurrent collaterals terminating on the PC layer and terminal boutons on neighbor PC somata (**Figure 2C**). We confirmed that these boutons made synapses, since they co-localized with the synaptic vesicle glycoprotein, synaptophysin (**Figure 2D**). With this tool at hand we proceeded to analyze numerous PC boutons from both WT and CB–/– mice in order to determine their spatial distribution patterns in the presence/absence of CB.

### ABSENCE OF CB INDUCES A CHANGE IN NUMBER AND VOLUME OF PRESYNAPTIC TERMINALS OF PC COLLATERALS

Dendrites and the main axon of PC are aligned in an almost perfect sagittal plane with minimal transversal deviations (Chan-Palay, 1971; King and Bishop, 1982). This is also the case for the recurrent collateral arbors that are entirely confined to very thin optical stacks from the confocal reconstructions of single biocytin-loaded PC, being the thickness of this stack generally <50 μm. This planar disposition of recurrent collaterals enabled us to examine confocal projections of PC in order to test whether morphological differences existed with respect to axon collateral synapses between WT and CB–/– mice.

**FIGURE 3:**
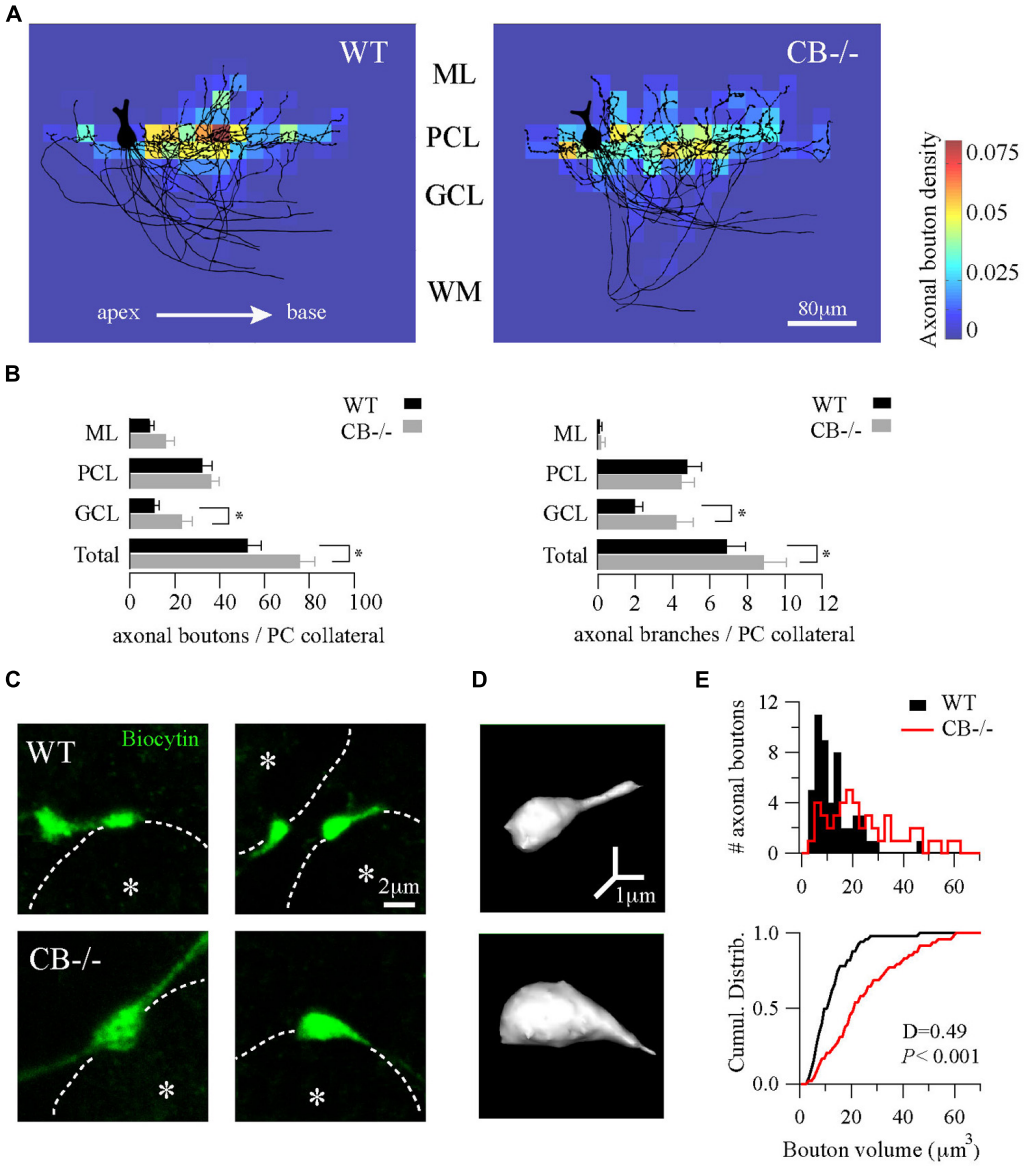
**Homeostatic changes in presynaptic boutons of PC recurrent collaterals of CB–/– mice. (A)** Axonal bouton density of PC collaterals in the cerebellar cortex. Biocytin-loaded PC were confocally imaged and superimposed for analysis of bouton distribution (*n* = 10 per genotype, PN18–25, see Materials and Methods). Somata were aligned (only one soma is shown, left) and axonal plexuses were oriented in the direction of the main PC axon pointing toward the DCN (right). As indicated by the white arrow, axonal plexuses ramified from the apex to the base of the cerebellar lobule. Heat maps were built and the number of boutons per area was calculated as bouton densities (boutons/μm^2^). Red and blue colors indicate maximal and minimal density of boutons, respectively. Axonal boutons from WT and CB–/– PC showed a similar spatial distribution by targeting more the PC layer and by respecting the apex-to-base orientation. **(B)** Pooled data for boutons and branches per PC collateral in the three layers of the cerebellar cortex from PC in A. CB–/– PC showed an increase in total number of boutons and branches compared to WT PC. This difference was essentially due to an increase in GCL boutons and branches without significant changes either in PCL or in ML. **(C)** Confocal projections of biocytin-loaded boutons terminating on neighbor PC somata (dotted white line). White asterisks show the half-radius of the targeted PC soma center. Note the size increase of CB–/– terminal boutons when compared to WT ones. **(D)** 3D-projections of the examples in C (right panels) corroborated the increase in volume. **(E)** Histogram for bouton volume values and the cumulative distributions showed a statistically significant difference between CB–/– compared to the WTs PC boutons. *P < 0.05, Student’s *t*-test.

We compared the distance to the first branch point, the angle that gave rise to the collateral and the total length of the collateral arbor; no statistically significant differences existed between genotypes (**Table 1**). Second, we evaluated the orientation/distribution of recurrent collateral arbors and boutons. For this we superimposed somata of biocytin-loaded PC and oriented their main axons toward the white matter, in order to align cortical layers (black traces in **Figure 3A**, *n* = 10 cells per genotype). Independent of the genotype, the main axons pointed in the direction of the DCN (to the base of the lobule) with their recurrent collaterals also pointing toward that direction. None to very few of recurrent collaterals returned in the opposite direction, *i.e.,* in the direction toward the apex of the lobule. This indicates that CB deletion in CB–/– mice did not affect the spatial orientation either of the principal PC axon or their recurrent collaterals. This is in line with a previous report demonstrating that in connected PC pairs a majority of PC collaterals (>80%) projected from the apex to the base of the lobule in both genotypes (Bornschein et al., 2013).

**Table 1:** Morphological measurements of axonal recurrent collaterals from WT and CB–/– mice.

*n*	WT 10	CB–/– 10	*P*
Distance until first branch (μm)	134.68 ± 14.61	134.02 ± 25.6	NS
Angle deviation of axon collateral (°)	145.4 ± 3.51	141.2 ± 6.26	NS
Total length collateral arbor (μm)	807.8 ± 81.15	950.19 ± 112.71	NS

*The three values presented in the table correspond to the distance from the axon hillock to the branching point, the angle of the collateral with respect to the main axon path and the length of the entire collateral branches from the branching point, respectively (from mice aged between PN18–PN25). Data are presented as mean ± SEM. No significant differences (NS) were observed between the two genotypes (Student’s *t* tests).*

Next, we calculated the bouton density on the superimposed PC per area represented as heat color maps shown in **Figure 3A** (for details, see Materials and Methods). In both genotypes the highest bouton densities (warm colors in the heat map) were seen to the right of the parental somata, *i.e.,* in the direction to the base of the lobule. A high prevalence of boutons within the PC layer was evident in comparison to the other layers. This indicates that the main postsynaptic targets of PC collaterals in either genotype were neighbor PC somata located approximately 20–80 μm away from the parental soma. However, the total number of boutons per collateral was increased in CB–/– mice compared to WT animals (**Figure 3B**, left panel). This difference was mostly due to an increase in GCL boutons (*P* < 0.05) that was accompanied by an increase in the number of branching points in the GCL of the cerebellar cortex (**Figure 3B**, right panel).

Finally, we focused on the morphology of boutons located on neighbor PC somata to explore subtle presynaptic changes in PC–PC synapses resulting from CB deletion. On images of z-stack projections of these boutons an increase in the size of CB–/– boutons compared to WT ones was observed (**Figure 3C**). A 3D-reconstruction pointed toward an increase in volume (**Figure 3D**), which was confirmed by counting the number of voxels per bouton. The volume distribution histograms showed a right-shift indicative of bigger values for CB–/– boutons compared to the WT boutons, also evidenced in the cumulative distribution curve shown in **Figure 3E**. In summary, these results imply that a likely homeostatic program of morphological axonal remodeling is induced in the absence of CB. This strongly hints toward changes at the subcellular level possibly in order to cope with the altered Ca^2+^ transients in CB–/– presynaptic PC terminals reported before (Bornschein et al., 2013).

### SUBCELLULAR ADAPTATIONS OF PC–PC SYNAPSES IN CB–/– MICE

**FIGURE 4:**
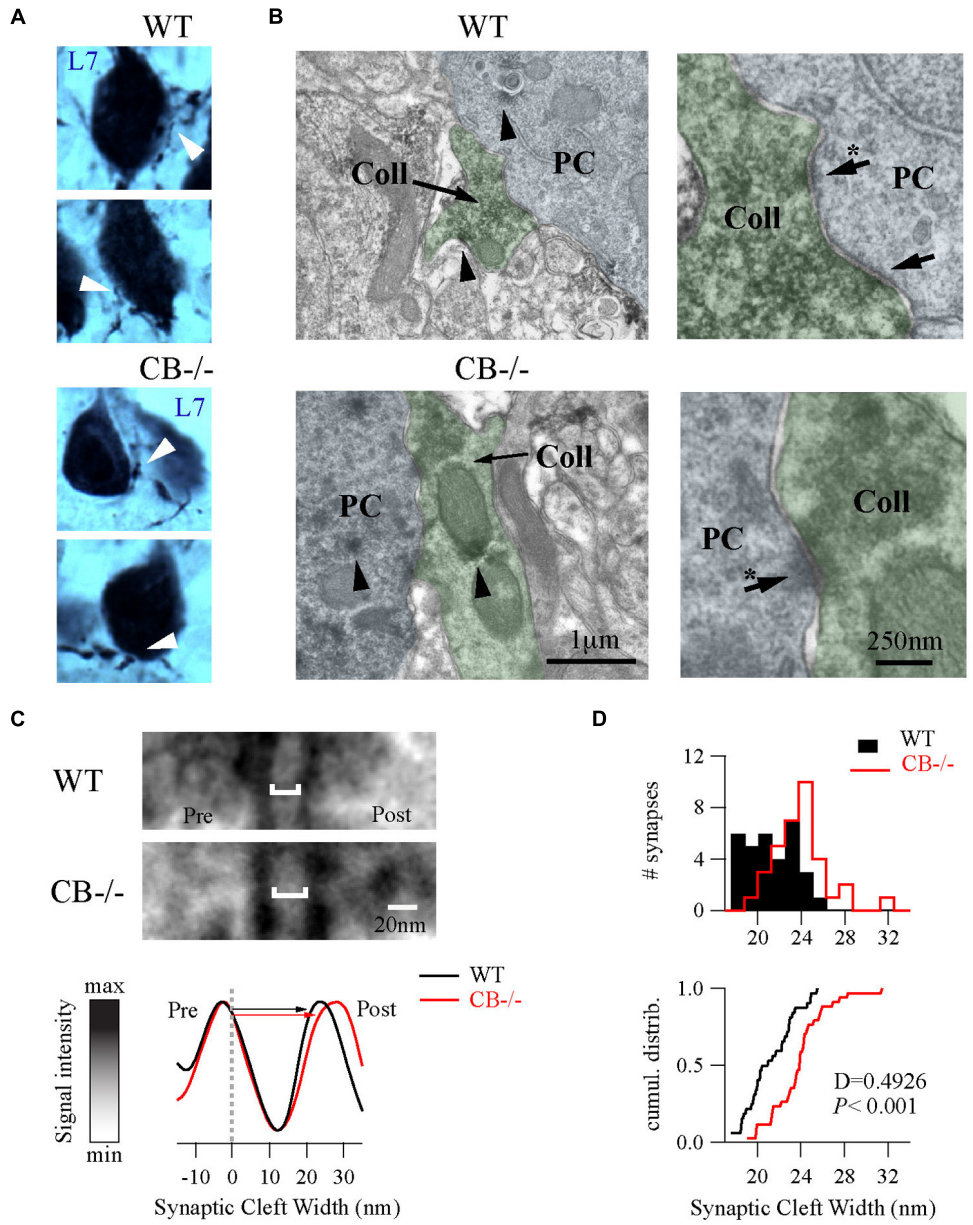
**Ultrastructural changes of PC–PC synapses in CB–/– mice. (A)** L7-immunolabelings were used to visualize axonal boutons on PC somata (white arrowheads) in both WT and CB–/– mice (PN20). WT boutons exhibited a smaller size than those from CB–/– mice. **(B)** Electronmicrographs of PC somata (blue background) and their presynaptic PC collateral boutons (green background) from both genotypes were recognized by their L7^+^ labeling (arrowheads) at X 30,000 magnification. Right panels are zoomed regions to better distinguish AZs (arrows). **(C)** Zoomed region of AZs from **(B)** (indicated by arrows with an asterisk on the right panels) showed an enlargement of synaptic cleft width in the CB–/– synapses compared to WT synapses (top). The line analysis method to properly measure synaptic cleft widths (bottom). Averages of five lines including pre/postsynaptic densities per synapse were normalized to peak/valley (see Materials and Methods). **(D)** Histograms and cumulative distributions showed that in CB–/– PC–PC synapses the right shift in the histogram (upper part) and cumulative distribution plot (lower part) indicate larger synaptic cleft values at CB–/– PC–PC synapses.

To further explore putative changes at the subcellular level of PC–PC synapses from both WT and CB–/– mice (PN20), we used EM to visualize presynaptic terminals onto PC somata and to determine their ultra-structural profile. Evidently when analyzing CB–/– mice, CB immunohistochemistry could not be used as a means to identify presynaptic terminals. To circumvent this problem we used the L7 protein (also named PCP-2), a marker exclusively localized within PC, expressed in all neuronal compartments and detected from early stages of neuronal maturation (Oberdick et al., 1990; Berrebi and Mugnaini, 1992). Moreover the L7 gene promoter has been widely used to efficiently direct targeted gene expression to PC (Smeyne et al., 1995) and L7 protein expression is observed throughout the PC cytosol. At the light and electron microscopic levels, L7 was visualized on somata and axonal terminals of PC from both genotypes (**Figure 4A**). Of note, L7^+^ boutons surrounding the PC somata were thinner in WT mice compared to CB–/– animals, which confirmed our previous observations obtained in single biocytin-loaded PC (**Figure 3C**).

As shown in **Figure 4B**, we identified PC–PC synapses by a detailed inspection of the somatic PC plasma membrane in order to localize L7^+^ boutons with symmetrical synaptic densities and pleomorphic synaptic vesicles as previously reported (Larramendi and Victor, 1967; Chan-Palay, 1971). The number of AZ per bouton was rather small (in the order of 1 – 3) and similar when comparing WT (1.19 ± 0.08; *n* = 32 from 2 mice) *vs*. CB–/– synapses (1.38 ± 0.1, *n* = 35 from 2 mice, *P* = NS). However, the length of individual AZ was 23% larger at CB–/– synapses (292.08 ± 15.89 nm, *n* = 35) compared to AZ of WT synapses (237.01 ± 14.43 nm, *n* = 32, *P* < 0.05). We also measured the synaptic cleft width between pre-post synaptic membranes of those synapses by using a newly developed method aimed to standardize the measurements and to maximally reduce experimenter bias (**Figure 4C**; also see Materials and Methods and Figure S1). Interestingly, we found that CB–/– cleft widths were bigger than WT ones (23.8 ± 0.43 nm *vs.* 21.17 ± 0.39 nm, respectively; *P* < 0.001; **Figure 4D**). To determine whether variances are equal or not within each group we performed homoscedasticity tests. In this test one calculates first the ratio of the largest to the smallest of the sample variances within one group (F_max_). If this ratio is bigger than unit, then one calculates what values F_max_ can attain (critical-F_max_) before one needs to reject the null hypotheses, which is that “variances are different” (see Materials and Methods). Neither for WT nor for CB–/– mice, F_max_ exceeded the critical-F_max_ values (1.19 *vs.* 2.46 for WT and 1.47 *vs.* 3.72, for CB–/–). This corroborated that synaptic cleft width values were homogeneous within each genotype and are in support that CB deletion in CB–/– mice induced a steady and constant change in the pre-post synaptic distance, which is assumed to have an impact on released GABA reaching the postsynaptic PC somata and thus on PC–PC IPSC characteristics (Bornschein et al., 2013).

**FIGURE 5:**
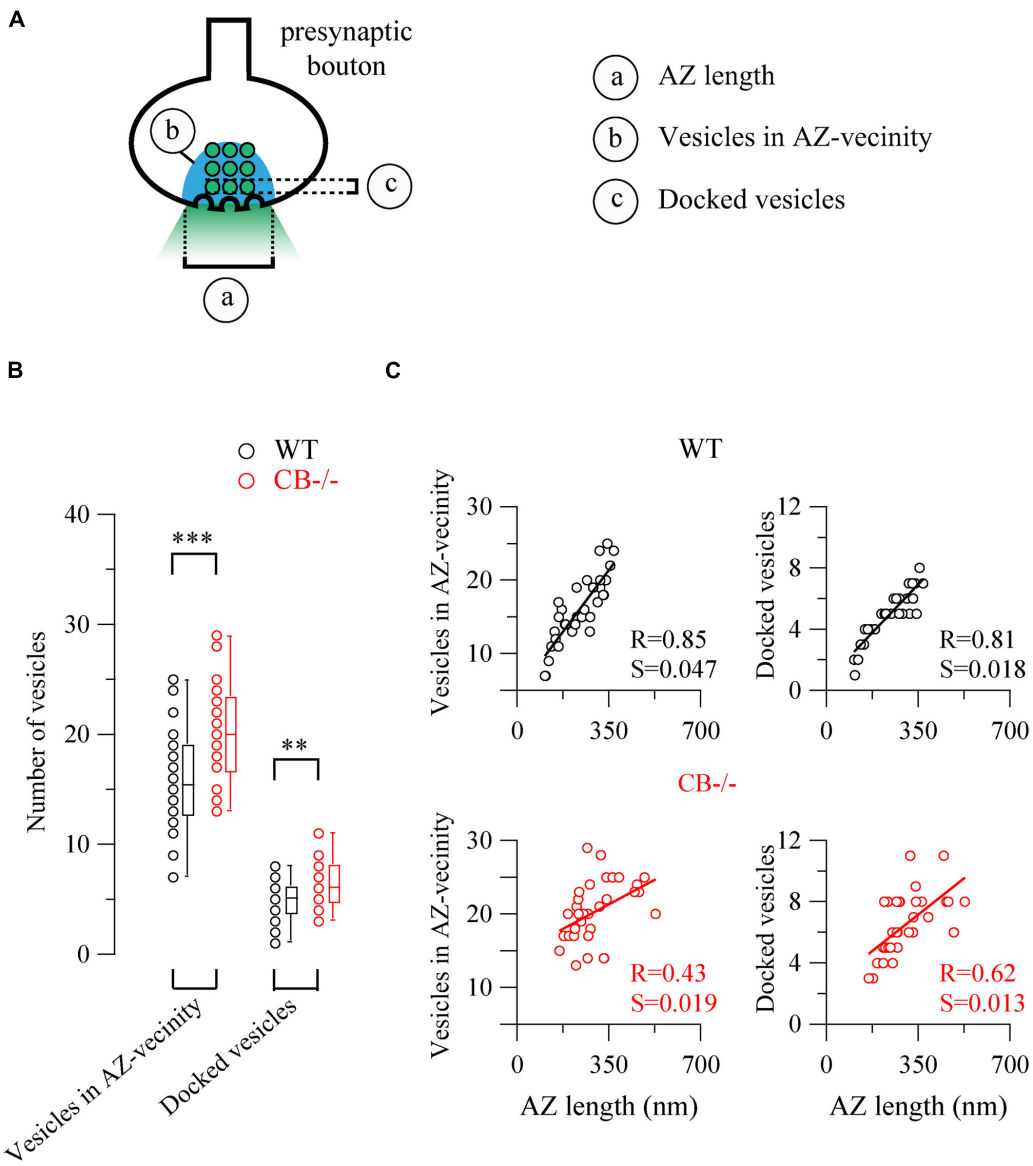
**The number of synaptic vesicles scales linearly with AZ length in both WT and CB–/– presynaptic boutons. (A)** The scheme shows the two approaches to count vesicles within a bouton in respect to the AZ. Vesicles in the “AZ vicinity” are considered as those located in an area covered by a half-circle with the diameter of the AZ length (blue region). Vesicles localized at a distance <10 nm from the plasma membrane of the AZ were counted as “docked vesicles.” **(B)** Pooled data from the two defined regions. CB–/– boutons contained more vesicles in the zone defined as “AZ vicinity” as well as docked vesicles per AZ. **(C)** Comparison between vesicle numbers and AZ length for each bouton. A strong linear relationship between AZ length and either docked or associated vesicles strongly indicated that the AZ length dictates the number of vesicles immediately ready to be released as well as those belonging to the pool for slower release. Statistical differences were calculated by Student’s *t* tests (**P < 0.01; ***P < 0.001). A 2-tailed test of significance was used for linear correlations (Pearson correlation) in **(C)** showing *P* values < 0.001 with the exception of *P* < 0.01 for the right bottom panel. R, coefficient of determination; S, slope.

Based on the observed changes in AZ length in CB–/– mice, we determined the vesicle number within this region, since it constitutes the site of synaptic vesicle clustering, docking and transmitter release (Rizzoli and Betz, 2005). Vesicles were counted in (*i)* the vicinity of the AZ, a region defined as a half circle with a diameter of the AZ length and (*ii*) in proximity of the AZ membrane, the latter representing the docked vesicle population (**Figure 5**). In both compartments, the number of vesicles was higher in CB–/– presynaptic terminals compared to WT, however, the overall vesicle density was similar in both genotypes: 8.97 ± 0.82 *vs.* 7.21 ± 0.58 vesicles per 100 nm^2^ for WT and CB–/– synapses, respectively (*P* = NS). This indicates that the number of vesicles was proportional to the AZ length, irrespective of the genotype and the vesicle/AZ length relationship is shown in **Figure 5C**. The vesicle number depended linearly on the AZ length, as it has been previously reported also for glutamatergic synapses (Holderith et al., 2012). Thus, the higher number of docked vesicles in PC–PC CB–/– synapses is in line with the observation of bigger *q* quanta values previously recorded in PC–PC CB–/– synapses (Bornschein et al., 2013). Of note the coefficient of determination (R) was smaller in CB–/– synapses compared to WT synapses, for both, the docked vesicles and the ones in the AZ vicinity. This might be an indication of a less tight organization of vesicles in the CB–/– presynaptic compartment and that potentially more vesicles were available for GABA release. This in turn, could underlie the reported increase in *p*. Thus, our results indicate that the absence of CB also affects the distribution/organization of vesicles at this synapse, likely also affecting the exocytotic machinery and consequently GABA release properties. Interestingly, in line with our results, an increase in the amount of Ca^2+^ entering the presynaptic terminal, *e.g.,* via Ca_V_2-type Ca^2+^ channels is often paralleled by an increase in the AZ size and the size of the ready-releasable pool of vesicles (Frank, 2014); other reported presynaptic HSP mechanisms include changes in *p* (Murthy et al., 2001).

## DISCUSSION

The brain is characterized by an extraordinary degree of plasticity, however, controlled by the presence of homeostatic signaling systems to keep the excitatory and inhibitory activity (E/I balance) within a “physiological” range thus allowing for a stable communication between neurons. From the viewpoint of information transfer at synapses, it is evident that synapses are under a bidirectional homeostatic control (Vitureira et al., 2012). By the process of HSP, neurons may modulate their excitability, firing properties and short- and/or long-term synaptic modulation in response to changes in activity in either direction, *i.e.,* as the result of increased or decreased net activity (Lee et al., 2014). Given the importance of Ca^2+^ ions in the process of synaptic transmission, both pre- and postsynaptically, an involvement of many components of the Ca^2+^-signaling toolkit (Berridge et al., 2003) in essentially all forms of synaptic plasticity has been reported (Cavazzini et al., 2005). Examples include presynaptic high-voltage activated Ca_V_2-type Ca^2+^ channels that gate forms of HSP. Weakened synapse activity leads to increased influx through Ca_V_2 channels, while enhanced influx via Ca_V_1-type channels elicits homeostatic adaptation by removal of postsynaptic excitatory receptors (for details, see Frank, 2014). The entity of all molecules that build the network of Ca^2+^ signaling components, and that are involved in their own regulation as to maintain physiological Ca^2+^ homeostasis resulting in phenotypic stability is named the Ca^2+^ homeostasome (Schwaller, 2009, 2012a).

The cerebellum represents a model system to investigate synaptic plasticity and the involved Ca^2+^-dependent processes, both accessible to experimental (reviewed in Lamont and Weber, 2012) and modeling approaches (Achard and Schutter, 2008). This highly repetitive structure consisting of relatively few distinct elements and a rather stereotyped wiring pattern, has allowed to investigate the role of proteins implicated in Ca^2+^ signaling in the various forms of plasticity. While rather much attention was given to systems implicated in Ca^2+^ entry from the extracellular side and to release mechanisms from internal stores, as well as systems leading to a decrease in [Ca^2+^]_i_, studies on the role of intracellular Ca^2+^-binding proteins in synaptic plasticity in the cerebellum are rather underrepresented. The “fast” buffer CB and the “slow buffer” PV are expressed in PC and PV additionally in stellate and basket cells. The effects that the absence of these proteins entail in PC and MLIs have been described in detail, both at the functional level (Schmidt et al., 2003; Collin et al., 2005; Franconville et al., 2011; Bornschein et al., 2013; reviewed in Schwaller, 2012b), but also at the level of PC morphology (Vecellio et al., 2000; Chen et al., 2006). The increased spine head volume and the longer spine shafts on CB–/– PC dendrites had been discussed as a HSP mechanism possibly contributing to unaltered LTD in CB–/– mice, although dendritic and spine [Ca^2+^]_i_ dynamics were altered in the absence of CB (Barski et al., 2003). With respect to the presynaptic function of CB, previous studies on recurrent PC axon collaterals forming synapses on neighboring PC (Orduz and Llano, 2007) have lead to several unexpected findings in CB-deficient mice (Bornschein et al., 2013). Although [Ca^2+^]_i_ amplitudes evoked by 10 APs delivered at 200 Hz were clearly larger in PC boutons of CB–/– PC, the evoked IPSC in the postsynaptic PC was unaltered and also PPF characteristics was unchanged in PN7–PN12 mice. This is at an age when CB expression levels have not yet attained adult levels and based on our results were estimated to be in the order of 50% compared to values in PN18–PN25 mice (**Figure 1**). This relates to a CB concentration in the range of 50 – 180 μM, since CB levels in PC of adult mice were reported to be in the range of 100 – 360 μM (for details, see Schmidt, 2012). Based on a modeling approach, the authors proposed that slow Ca^2+^ unbinding from the sensor for transmitter release was the main determinant for PPF dynamics and thus independent from CB (Bornschein et al., 2013). This is in contrast to the synapse between cortical CB-expressing multipolar bursting (MB) cells and pyramidal cells, where rapid Ca^2+^ buffer (CB) saturation resulted in a decreased IPSC amplitude of the first response and increased PPF by this buffer effect, a process termed “facilitation by Ca^2+^ buffer (CB) saturation (Maeda et al., 1999; Blatow et al., 2003) or “pseudo-facilitation” (Neher, 1998; Rozov et al., 2001; Zucker and Regehr, 2002). These experiments had been carried out in acute slices after washout of CB via the patch pipette (representing the CB–/– situation) and reverted by loading of terminals with CB or BAPTA, in both cases not allowing for homeostatic plasticity mechanisms to come into play. Additional experiments showed that PPF at MB cell terminals depend on Ca^2+^ influx rather than on the initial *p*.

In PC–PC connections in CB–/– mice, both *p* and *q* are increased, findings that hinted toward an induction of likely homeostatic mechanisms possibly aimed at restoring IPSC amplitudes and STP as seen in WT terminals. Both parameters, *p* and *q*, strongly correlate with bouton volume (Schikorski and Stevens, 1997; Murthy et al., 2001) and peak amplitude of presynaptic [Ca^2+^] transients has been seen positively correlated with the AZ area (Holderith et al., 2012). Such a volume increase was also evident in PC boutons from CB–/– mice (**Figure 3**); moreover an increase in bouton volume is also associated with a larger AZ, paralleled by an almost identical increase in the size of the PSD (Murthy et al., 2001). While a larger AZ encompasses more docked vesicles (Holderith et al., 2012), which is in line with an increase in the magnitude of *q*, such a straightforward correlation appears not to hold true for the *p*. Several possibilities were considered to explain differences in *p* at CF and PF synapses, despite the number of docked vesicles being similar (Xu-Friedman et al., 2001). Alterations in the priming process of docked vesicles or in the phosphorylation state of proteins implicated in the exocytotic machinery, but also changes in the Ca^2+^ signal resulting from changes in Ca^2+^ influx or Ca^2+^ buffering were discussed to affect *p*. An example of the former was observed in boutons of hippocampal neurons, where an increase in Ca^2+^ influx by TTX pretreatment increased *p*, while the number of readily releasable vesicles was only marginally reduced (Zhao et al., 2011).

Although EM 3D-reconstructions from serial ultrathin sections could permit a most accurate view of the ultrastructure (Pierce and Mendell, 1993), we considered the AZ/PSD length of recurrent PC axon collaterals as a proxy measure for the area of these appositions, *i.e.,* we assumed these contacts to be circular in shape. Accepting these shortcomings, the area of a PC–PC contact was calculated to be approximately 50% larger in CB–/– terminals. Studies that reported on the width of the synaptic cleft found the cleft width to show extremely little variation (SD in the order of <5%) for a given synapse type (Jing et al., 2004; Xu and Zhang, 2006; Xu et al., 2013a,b; Han et al., 2014). Changes in cleft width are most often the result of experimental manipulations including OGD, “metal-poisoning” by lead and aluminum (Jing et al., 2004; Lushnikova et al., 2011; Han et al., 2014), but were also observed in genetic models, *e.g.,* CNTNAP4-KO mice (Karayannis et al., 2014). Of note cleft width changes appear to be independent from changes in the length/area of the AZ. After 60 min OGD, AZ area increased by 58% (Lushnikova et al., 2011) and also the volume of the presynaptic terminal was augmented. In the other models, where cleft width was increased, the length of the AZ was either unaltered, as in the case of estradiol benzoate treatment (Xu et al., 2006) or even reduced as in the cases of bisphenol-A-mediated inhibition of synaptogenesis (Xu et al., 2013a,b) and exposure to either lead (Han et al., 2014) or aluminum (Jing et al., 2004).

**FIGURE 6:**
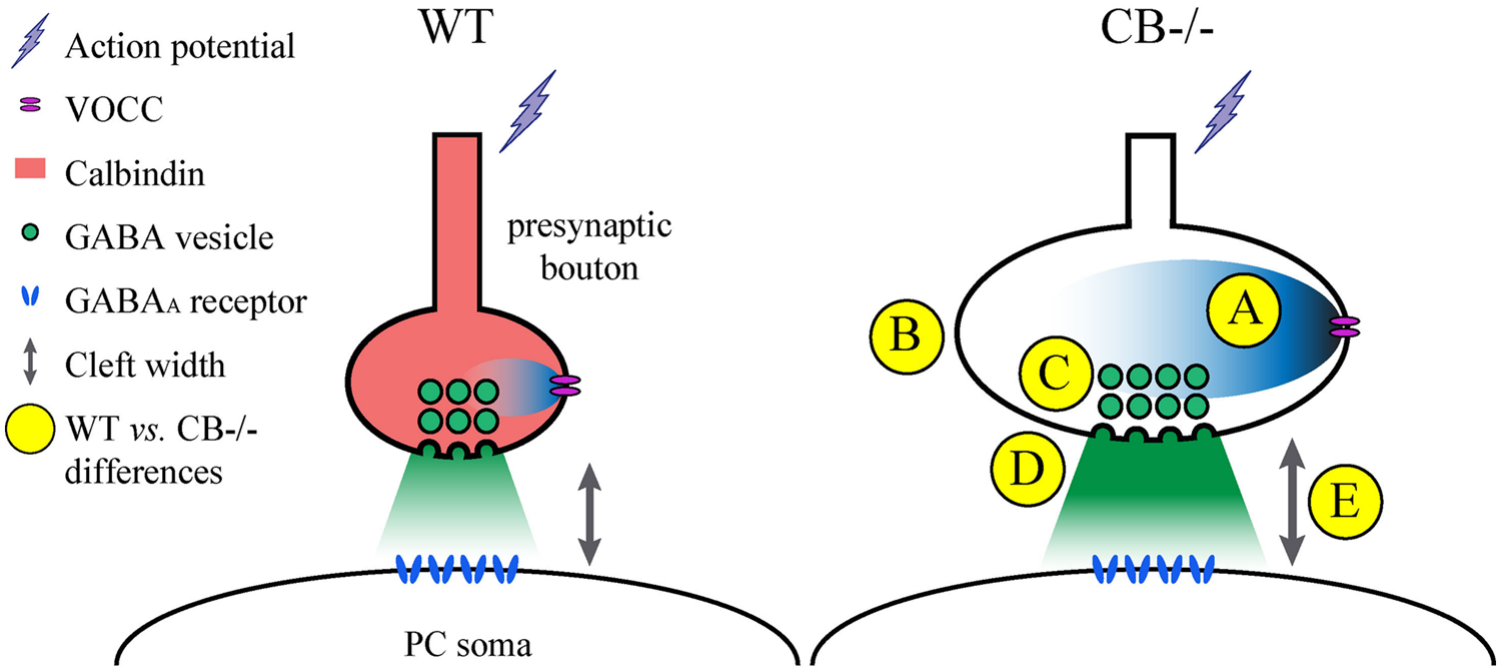
**Summary of the observed and proposed alterations at PC–PC synapses due to CB deletion. (A)** An AP arrives at the presynaptic terminal and causes opening of voltage-operated Ca^2+^ channels (VOCC). The resulting intracellular rise in [Ca^2+^]_i_ is shaped by the presence of CB (Bornschein et al., 2013), which determines the efficacy of synaptic transmission. In the absence of CB, the maximal amplitude of Ca^2+^ transients is increased in CB–/– presynaptic boutons compared to WT controls. **(B)** To possibly reduce the impact of the Ca^2+^ transients on the amount of GABA released into the synaptic cleft, the volume (and surface) of presynaptic boutons of CB–/– mice is increased. **(C)** This is also reflected by a stretching of the AZ length, which is accompanied by a proportional increase in the number of ready releasable vesicles. **(D)** This might explain the enhancement in synaptic efficacy characterized by increased *p* and *q* previously observed at CB–/– PC–PC synapses (Bornschein et al., 2013). Yet postsynaptic PC responses with respect to IPSC mean amplitudes or STP are indistinguishable between PC pairs from WT or CB–/– mice (Bornschein et al., 2013). **(E)** An enlargement of synaptic cleft width and AZ length resulting in an approximately 70% larger volume of the synaptic cleft might reduce the GABA concentration reaching the postsynaptic GABA receptors finally resulting in unaltered IPSC amplitudes and PPF characteristics at CB–/– PC–PC synapses. It can’t be excluded that other mechanisms, *e.g.,* subtle alterations in the subunit composition of GABA_A_ receptors might also contribute to this likely homeostatic mechanism of plasticity.

One of the problems with measuring cleft width on EM images is the procedure used to accurately measure this parameter. Different approaches have been chosen. Here we have compared a manual and a standardized procedure for measuring cleft width. Although absolute values are different when applying the 2 methods (Figure S1), synaptic cleft width was clearly larger in CB–/– PC–PC synapses. The volume of the synaptic cleft was then estimated to be a cylinder defined by the surface area of the AZ/PSD and the height of the synaptic cleft; this volume was increased by 70% in CB–/– PC–PC synapses. Thus, it is foreseeable that the increased volume would suffice to diminish the GABA concentration at the surface of the postsynaptic side and thus IPSC amplitude, as previously modeled for excitatory synaptic transmission (Wahl et al., 1996). This, in turn may lead to a postsynaptic response (IPSC) of similar magnitude as observed in WT PC (**Figure 6**).

Besides the changes in the architecture of PC–PC contacts in CB–/– mice, the number of boutons per PC collateral was increased. Studies on cerebellar synapse formation during postnatal development have mostly focused on the formation of PF–PC synapses and CF–PC synapses. In the mouse the process of CF–PC synapse formation/elimination is a multistep process consisting of initially multiple innervation of a PC soma by several CF (PN3) followed by functional differentiation (PN7). At this time point also PF synapses, as well as MLI synapses start to form. The later stages consist of CF synapse translocation essentially to PC dendrites (PN9) and an early (PN8–PN11) and late (PN12–PN17) phase of CF elimination, resulting in a single CF innervating each PC (reviewed in Hashimoto and Kano, 2013). What is currently known about the temporal development of PC–PC synapses? The PC intracortical plexus starts to form during the first postnatal week characterized by sprouting with a maximal branching reached around PN6 (Gianola et al., 2003). PC collateral start to form synapses around PN7, just at the time when functional differentiation of CF synapses is finished. CF synapse formation is considered to be the first step during cerebellar synaptogenesis (Larramendi, 1969). The second postnatal week is then exemplified by augmented structural plasticity consisting of trimming of collateral branches to less than half compared to PN6 and in remodeling of terminal arbors. Excess fibers that make up this tangled plexus disappear largely around PN20, but stable long-term connections are preserved from PN15 onward in rats (Gianola et al., 2003). Paired PC–PC recordings in young rodents revealed a decrease in the probability of connections between the first and second PN weeks compared to the third PN week: 26% at PN4–14 (Watt et al., 2009) and 10% at PN15–19 (Orduz and Llano, 2007). This decrease may be viewed as an advantage of CF–PC somatic innervation *vs.* PC–PC connections during postnatal development (Larramendi, 1969). Of interest, a mathematical model recapitulating *in vivo* recordings of cerebellar oscillations in adult rats, in which PC–PC connections are fully operational, requires a connection probability of 20% between PC in order to reproduce this oscillatory behavior (de Solages et al., 2008). Thus, the higher number of axonal branches and the increased number of boutons in the GCL of CB–/– mice might, in part, explain the strong 160-Hz oscillations observed in adult CB–/– mice that are essentially absent in WT mice (Servais et al., 2005). Thus, one might speculate that the situation of PC collaterals in CB–/– mice resembles the situation of early development, when collaterals are more numerous, inhibition via MLI has not taken place yet, resulting in synchronization in the sagittal plane in the form of traveling waves (Watt et al., 2009).

We reasoned that the time window of the formation of PC–PC synapses would be the same as for spine development, *i.e.,* in conjunction with the functional maturation of the cerebellum. In developing hippocampal neurons during the period of rapid synaptogenesis *in vitro*, the blocking of spike activity by tetrodotoxin (TTX) reduces the density of inhibitory synapses, both onto glutamatergic and GABAergic target neurons, without affecting density of glutamatergic synapses (Hartman et al., 2006). Yet in other experimental settings using cortical or hippocampal cultures, also the density of excitatory synapses was found to be increased after TTX treatment (Wierenga et al., 2006) and decreasing neuronal circuit activity in hippocampal cultures resulted in an increase in the number of connected neuron pairs (Nakayama et al., 2005). Thus, both excitatory as well as inhibitory synapses are receptive for activity-dependent modulation by modifying synapse numbers. Here we propose that the increase in CB–/– PC synaptic boutons might be viewed as a means to increase inhibition onto close neighbor PC. The most prominent increase in axonal boutons was observed in the GCL and less in the PCL indicating that possibly axo-axonic and not axo-somatic inhibition might be increased. Thus, these morphological findings indicate that the output of an individual PC to the DCN is possibly more strongly controlled by neighbor PC. Another interesting point concerns the fact that myelin formation shapes cerebellar connections by removing excess collateral branches of Purkinje neurons (Gianola et al., 2003). In the case of CB–/– mice, the increased branching in the GCL, the layer in which more myelin is wrapped around the PC axons might be indicative of a permissive signal generating branching and possibly more synaptic contacts onto postsynaptic targets of PC axon collaterals, *e.g.,* on other PC axons, on interneurons or even on NG2^+^ progenitors (Boda and Buffo, 2014).

What are the consequences of the absence of CB with respect to cerebellar function and do the reported HSP mechanisms suffice to “prevent” a CB–/– motor phenotype? As mentioned above, LTD is not affected in CB–/– mice, while motor coordination and motor learning are impaired (Barski et al., 2003; Farré-Castany et al., 2007), as shown in the runway assay, and by measuring the optokinetic reflex OKR (Barski et al., 2003). This has, in part, been attributed to the presence of 160 Hz cerebellar oscillations recorded from alert CB–/– mice that are essentially absent in WT mice (Servais et al., 2005). These oscillations are the result of synchronous activity along the PF beam with a likely contribution of the recurrent PC axon collaterals (Maex and Schutter, 2005). Whether higher cognitive functions are impaired in CB–/– mice is currently unknown. A decrease in CB expression levels and/or CB-ir neurons has been reported in several neurological diseases including schizophrenia, bipolar disorder and autism spectrum disorders (Wondolowski and Dickman, 2013). It remains to be investigated whether CB–/– mice also show behavioral changes reminiscent of these pathologies.

## AUTHOR CONTRIBUTIONS

David Orduz, David Gall, Serge N. Schiffmann, and Beat Schwaller conceived and designed the experiments; David Orduz, Alain Boom, and Beat Schwaller performed research; David Orduz and Beat Schwaller analyzed the data; all authors interpreted the data; David Orduz and Beat Schwaller drafted the manuscript, all authors critically revised the manuscript and approved the final version of the manuscript.

## Conflict of Interest Statement

The authors declare that the research was conducted in the absence of any commercial or financial relationships that could be construed as a potential conflict of interest.

## ABBREVIATIONS

AP: action potential
AZ: active zone
CB: calbindin D-28k
CB–/–: calbindin knock-out mice
CF: climbing fiber
CV: coefficient of variation
DCN: deep cerebellar nuclei
EM: electron microscopy
GABA: gamma amino butyric acid
GCL: granular cell layer
HSP: homeostatic synaptic plasticity
IPSC: inhibitory postsynaptic current
LTD: long-term depression
LTP: long-term potentiation
ML: molecular layer
MLI: molecular layer interneuron
*p*: release probability
PC: Purkinje cell
PCL: Purkinje cell layer
PF: parallel fiber
PN: postnatal day
PSD: postsynaptic density
*q*: quantal size
STP: short-term plasticity.
