# Supplementary material for: Subcellular structural plasticity caused by the absence of the fast Ca2+ buffer calbindin D-28k in recurrent collaterals of cerebellar Purkinje neurons
Source: Front Cell Neurosci. 2014 Nov 5;8:364. doi: 10.3389/fncel.2014.00364 (PMC4220698; doi:10.3389/fncel.2014.00364)
Supplement: Supplementary file 1 [file Data_Sheet_1.PDF]

Supplementary Material

Subcellular structural plasticity caused by the absence of the fast Ca^{2+} buffer calbindin D-28k in recurrent collaterals of cerebellar Purkinje neurons

David Orduz [1]*, Alain Boom [2], David Gall [1], Jean-Pierre Brion [2], Serge N Schiffmann [1] and Beat Schwaller [3]*

[1] Laboratory of Neurophysiology, UNI, Université Libre de Bruxelles (ULB), Bruxelles, Belgium
*[2] **L**aboratory of Histology, Neuroanatomy and Neuropathology, UNI, Université Libre de Bruxelles (ULB), Bruxelles, Belgium*
[3] Anatomy, Department of Medicine, University of Fribourg, Fribourg, Switzerland

**** Correspondence:** David Orduz, Neurophysiology & New Microscopies Laboratory, INSERM U1128, Paris Descartes University, 45 Rue des Saints-Pères, 75006, Paris, France. e-mail: david.orduz-perez@parisdescartes.fr; Beat Schwaller, Anatomy, Department of Medicine, University of Fribourg, Route Albert-Gockel 1, CH-1700 Fribourg, Switzerland. e-mail: beat.schwaller@unifr.ch*

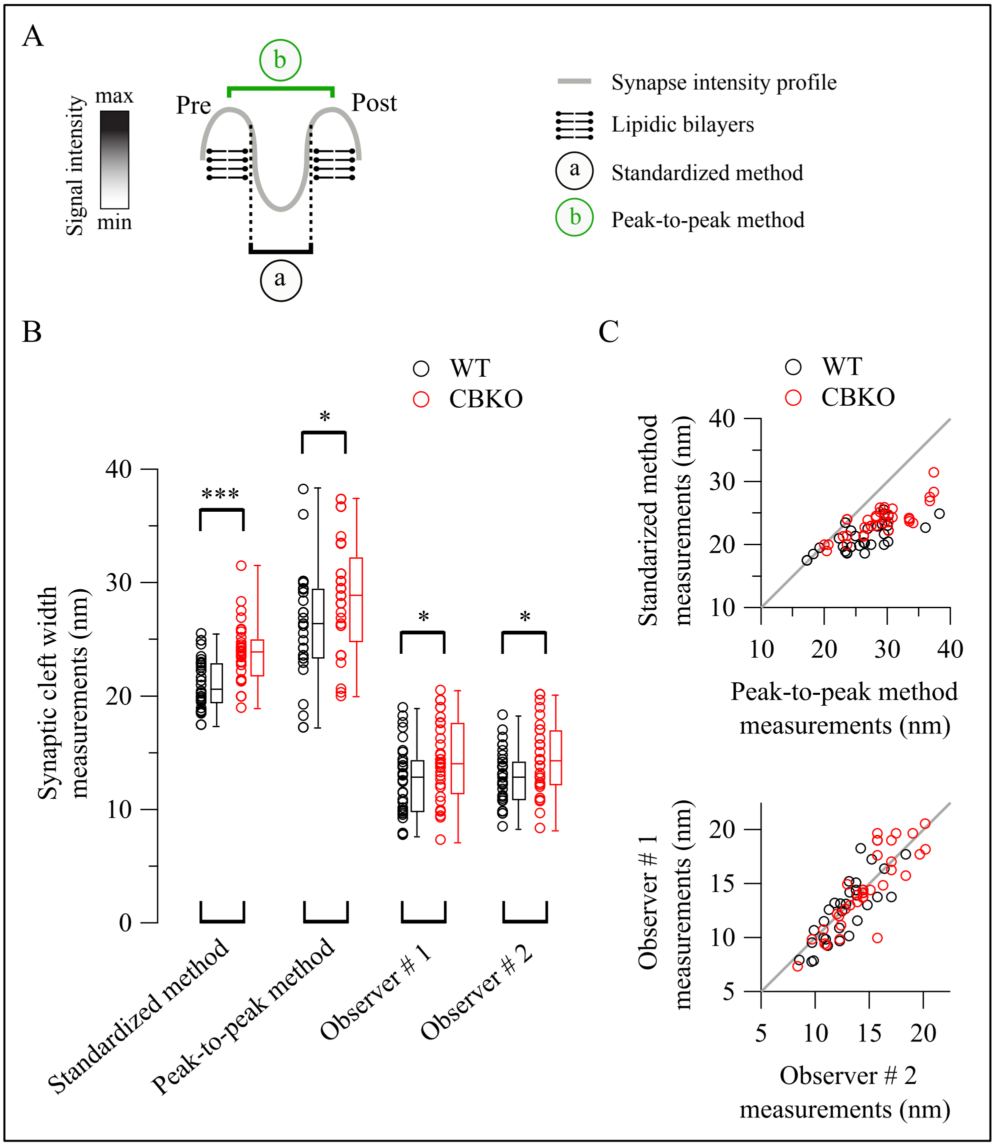

Supplementary Figure 1. A standardized method for accurate measuring synaptic cleft width in PC-PC synapses. (A) The cartoon illustrates the intensity profile for pre-to-postsynaptic membrane peak densities (maximal intensity signal). Note that the valley in the center (minimal intensity signal) corresponds to the "real synaptic cleft width" that doesn't include the electrodense lipid bilayers. Lower case letters indicate the boundaries for the two methods used to calculate the synaptic cleft width from intensity profiles (see methods). **(B)** Synaptic cleft width measurements and their box-to-whiskers graphs color-coded per genotype. Data obtained with the two methods and also by two-blind experimenters (Observers #1 and #2) are presented for comparison. **(C)** Comparison between measurements performed by standardized *vs.* peak-to-peak method (top) and between observers for the same synapses (bottom). When synaptic cleft widths from the same genotype were measured with the standardized method, its distribution presented smaller coefficient of variation

(CV) compared to the peak-to-peak method (0.32 *vs.* 0.42 respectively for WT synapses; and 0.32 *vs.* 0.4 respectively for CB-/- synapses) and the mean width values were smaller (21.17 ± 0.39 *vs.* 26.28 ± 0.83 for WT synapses; and 23.8 ± 0.43 *vs.* 28.73 ± 0.82 for CB-/- synapses). This confirmed that the standardized method catches the "real synaptic cleft width". Interestingly, this method increased the statistical significant difference between genotypes (P<0.001) compared to the peak-to-peak method (P<0.05), which corroborates its robustness to properly measure synaptic widths and to unravel subtle differences. On the other hand, values from blind-experimenters showed bigger CV values compared to the standardized method (0.48 and 0.42 for Observer #1 and #2 respectively for WT synapses; 0.49 and 0.45 for Observer #1 and #2 respectively for CB-/- synapses) and smaller mean values compared to the standardized method (12.73 ± 0.53 and 12.9 ± 0.51 for Observer #1 and #2 respectively from the WT synapses; 14.38 ± 0.6 and 14.66 ± 0.51 for Observer #1 and #2 respectively from the CB-/- synapses). This confirmed that the eye-inspection method likely underestimates the real synaptic cleft width and introduces a more substantial experimenter-dependent bias to the measurements. *P < 0.05; ***P < 0.001; Student's t test.
